# Impact of COVID-19 on patient experience of kidney care: a rapid review

**DOI:** 10.1007/s40620-023-01823-5

**Published:** 2023-12-20

**Authors:** Lucy Mackintosh, Paula Ormandy, Amanda Busby, Janine Hawkins, Ranjit Klare, Christina Silver, Maria Da Silva-Gane, Shalini Santhakumaran, Paul Bristow, Shivani Sharma, David Wellsted, Joseph Chilcot, Sivakumar Sridharan, Retha Steenkamp, Tess Harris, Susan Muirhead, Vicky Lush, Sarah Afuwape, Ken Farrington

**Affiliations:** 1 School of Life and Medical Sciences, University of Hertfordshire, Hertfordshire, UK; 2 University of Salford, Salford, UK; 3 UK Kidney Association, Bristol, UK; 4 Qualitative Data Analysis Services, Gillingham, UK; 5 East and North Hertfordshire NHS Trust, Stevenage, UK; 6 Kidney Care UK, Alton, UK; 7 Institute of Psychiatry, Psychology and Neuroscience, King’s College London, London, UK; 8 PKD Charity, London, UK; 9 Portsmouth Hospital, Portsmouth, UK; 10 Royal Free London NHS Foundation Trust, London, UK; 11 UCL Division of Medicine, University College London, London, UK

**Keywords:** Chronic kidney disease, Coronavirus, Kidney care, Patient experience

## Abstract

**Introduction:**

In March 2020, a pandemic state was declared due to SARS-COV-2 (COVID-19). Patients with kidney disease, especially those on replacement therapies, proved more susceptible to severe infection. This rapid literature review aims to help understand how the pandemic impacted patient experience of kidney care.

**Methods:**

It was conducted in accordance with Cochrane Rapid Review interim guidance. Search terms, ‘coronavirus’, ‘kidney care’, and ‘patient-reported experience’ and terms with similar semantic meaning, identified 1,117 articles in Medline, Scopus, and Worldwide Science. Seventeen were included in the narrative synthesis.

**Results:**

The findings were summarised into three themes: remote consultation and telemedicine (*n* = 9); psychosocial impact (*n* = 2); and patient satisfaction and patient-reported experience (*n* = 6). Patients were mostly satisfied with remote consultations, describing them as convenient and allowing avoidance of hospital visits. Anxieties included missing potentially important clinical findings due to lack of physical examination, poor digital literacy, and technical difficulties. Psychosocial impact differed between treatment modalities—transplant recipients expressing feelings of instability and dread of having to return to dialysis, and generally, were less satisfied, citing reduced ability to work and difficulty accessing medications. Those on home dialysis treatments tended to feel safer. Findings focused on aspects of patient experience of kidney care during the pandemic rather than a holistic view.

**Conclusions:**

There was little direct evaluation of modality differences and limited consideration of health inequalities in care experiences. A fuller understanding of these issues would guide policy agendas to support patient experience during future public health crises.

**Graphical abstract:**

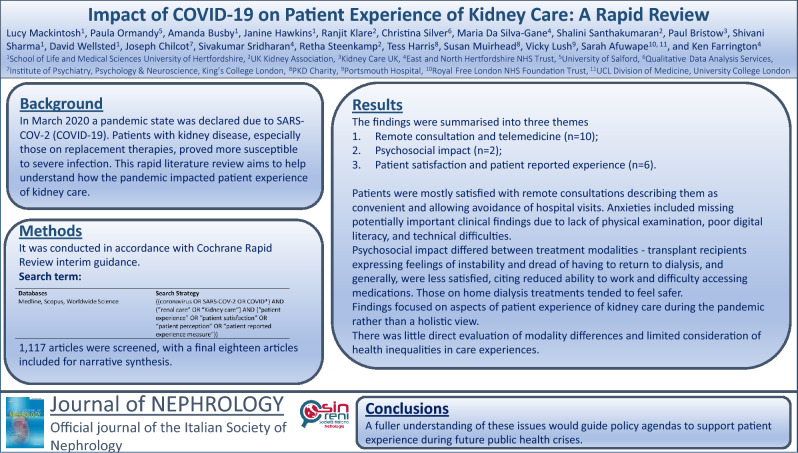

**Supplementary Information:**

The online version contains supplementary material available at 10.1007/s40620-023-01823-5.

## Introduction

Chronic kidney disease (CKD) affects around 10% of the population worldwide [1]. There is a higher prevalence in older individuals, women, and people of minority ethnic heritage. Chronic kidney disease is one of the leading causes of death worldwide [1]. In March 2020, the World Health Organisation (WHO) declared a pandemic state due to the outbreak of SARS-COV-2, later renamed Coronavirus Disease 2019 (COVID-19). COVID-19 is an acute respiratory syndrome resulting in fever, cough, loss of sense of smell, and in some cases severe pneumonia, organ failure and death [2]. Patients with kidney disease, especially those with advanced disease treated by dialysis or transplantation, have suppressed immune systems [3] and are at higher risk of developing more severe complications [4].

In the UK, prior to 2020, the National Health Service (NHS) was already operating with limited resources making it ill-prepared for the pandemic [5], like many healthcare systems around the world [6]. One study evaluating the economic impact of COVID-19 on healthcare facilities and services outlined that, internationally, personal protective equipment for healthcare workers, hospital equipment, and sanitary resources were in short supply [6].

In the US, all non-emergency surgeries and other procedures were cancelled to allow for staff and beds to be redeployed to wards treating individuals infected with COVID-19. Additionally, nearly all outpatient consultations were transitioned to telemedicine appointments [7]. With the increased cost for hospitals in the US associated with COVID-19, e.g., increased demand for personal protective equipment, and reduced income from cancelled surgeries, hospitals across the country faced grave financial strain [7].

Australia implemented similar changes even though case numbers for COVID-19 were relatively low. Routine appointments and surgeries were cancelled to prepare for an increase in hospitalisations related to COVID-19. Healthcare-seeking behaviour changed, with individuals showing a reluctance to visit primary care in-person (down 22.1% from 2019). Ambulance incidents also decreased (by 7.2%), as well as emergency department visits (13.9%) [8].

During the pandemic, UK healthcare changed dramatically to try to ensure delivery of safe care; this differed slightly between wave 1 of the pandemic (March 2020 to August 2020) and wave 2 (September 2020 to June 2021), with wave 2 providing easier access to COVID-19 swab testing, and hospital wards being better equipped to face the COVID-19 burden [9]. There was a rapid escalation of remote consultations replacing the traditional face-to-face. Those in need of in-person care experienced long delays and inaccessibility due to reductions of elective care, diversion of resources to acute care of patients with COVID-19, and chronic workforce shortages [5]. Patients with long-term conditions were disproportionately impacted in care experiences.

To investigate the impact of COVID-19 on the NHS, tweets posted between January 2018 and October 2020, by individuals with long-term health conditions based in the UK, were harvested to better understand the discourse around changes to healthcare delivery [10]. From 637 relevant tweets, five themes emerged; access to remote care (41.9%), quality of remote care (20.4%), anticipation of remote care (6.1.%), online booking and asynchronous communication (13.3%), and publicising changes to services or care delivery (25.1%). The proportion of positive tweets in relation to the quality of remote care was higher in the immediate period following the outbreak (March–May 2020) than following easing of the first lockdown (June–October 2020) [10].

Little research has addressed the impact of the pandemic on the experience of care of people with kidney disease. This rapid review was carried out to investigate this issue. The review forms part of a multi-phase study funded jointly by the British Renal Society and Kidney Care UK investigating the responses of kidney centres, changes in practice patterns and the experience of patients with kidney disease during the COVID-19 pandemic.

## Methods

### Aims and objectives

We aimed to address the following questions:

1. What was the impact of COVID-19 on patient experience of kidney care?
2. Which aspects of patient experience of kidney care were impacted most during COVID-19?

Our objective was to produce a narrative synthesis of the peer-reviewed literature to identify major themes describing patient experience of kidney care during the COVID-19 pandemic to help optimise future care, direct future research and plan provision during future severe civil disruption.

### Search strategy

The review was conducted in accordance with Cochrane Rapid Review interim guidance [11]. The search terms (see Table 1) were used aligned to the concept of COVID-19 and patient experience. The search terms were employed across all three databases- Medline, Scopus and worldwide science, optimising searches as per the structure of different databases. The search terms were decided upon by the study management group. This consisted of people receiving care for CKD including those receiving dialysis and those living with a kidney transplant, along with doctors, nurses, psychologists, and social workers involved in the care of people living with CKD, representatives of kidney patient charities, and researchers. The search strategy is depicted in Table 1.

**Table 1:**
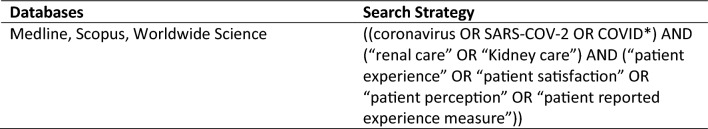
Search strategy

The search period was January 2019–August 2022. Searches were limited to papers published in English. Two authors (LM [1] and KF [2]) reviewed the papers based on title and abstract using a web-based tool for literature reviews (Rayyan) [12]. Reviewer one completed the initial inclusion of all potentially relevant articles. Reviewer two, using blind mode to conceal categorisation from reviewer one, checked 20% of identified papers for agreement. Conflicts were discussed and resolved, with reasons for choices given and referring to the eligibility criteria (below), following the process outlined by Cochrane [11]. Full manuscripts were obtained and screened in detail for inclusion.

### Inclusion/exclusion criteria

#### Inclusion criteria

Both quantitative and qualitative studies were included if they met the following conditions: conducted during COVID-19 (from March 2020 to August 2022), measuring patient experience and/or satisfaction and/or perception of kidney care and focussed on the views of people living with CKD. This included patients with stage 3–5 CKD and those receiving in-centre haemodialysis (ICHD) or satellite (Sat HD), home therapies [peritoneal dialysis (PD) and home haemodialysis (HHD)] and those who are living with a kidney transplant.

#### Exclusion criteria

Articles were excluded based on the following criteria: indirectly mentioning people living with CKD, and exclusively reporting on patients with kidney disease who had contracted COVID-19.

#### Data items and extraction

A data abstraction form was developed in MS Excel, containing the following data fields from each of the included studies: authors, date, country, title, and reference, aim, setting, number of individuals studied and stage of kidney disease/modality type (CKD, haemodialysis, PD, transplant recipient), instruments used and validation, method of data collection, main findings, and study limitations.

### Analysis and assessment of bias

A narrative synthesis was conducted following data extraction of the included articles. This consisted of a unification of the findings from multiple studies relying primarily on the use of words and text to summarise and to explain these findings in a harmonised manner [11]. This analysis aimed to describe the impact of COVID-19 on patient experience of kidney care, highlighting the aspects of kidney care mainly affected by the pandemic, and patient experiences of these changes. This analysis of data aimed to describe the impact of COVID-19 on patient experience of kidney care, highlighting the aspects of kidney care mainly affected by the pandemic, and patient experiences of these changes. Using the Mixed Methods Appraisal Tool (MMAT) [13] both reviewer one and two completed a risk of bias assessment on each article, with conflicts in ratings discussed.

## Results

### Paper identification

**Fig. 1:**
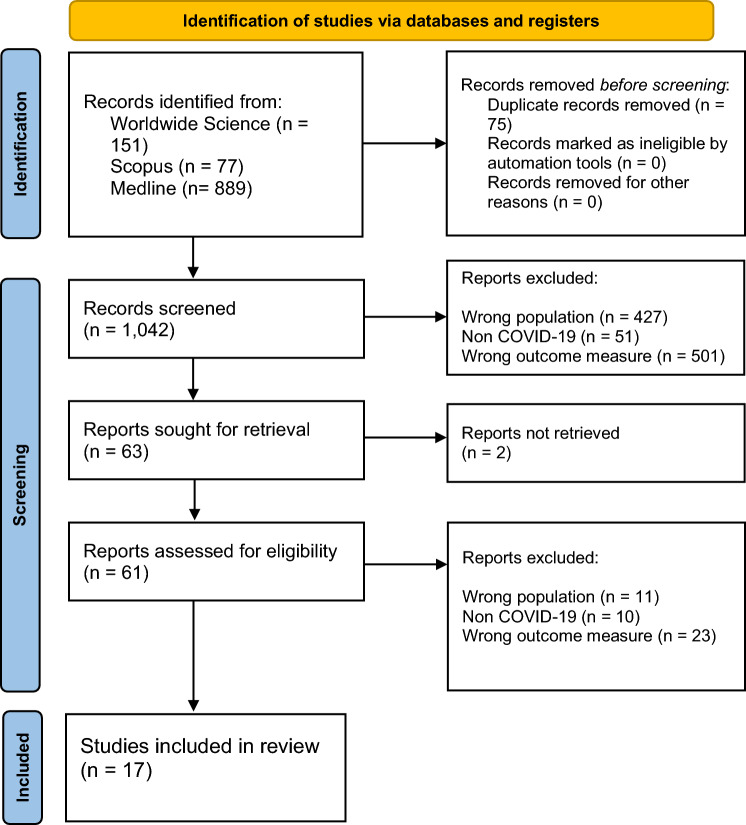
Preferred reporting items for systematic reviews and meta-analyses (PRISMA) flow diagram of the study [14]

The study flow diagram is shown in Fig. 1. A total of 1,117 articles were retrieved (Medline *n* = 889, Scopus *n* = 77, Worldwide Science *n* = 151). Duplicates were removed, resulting in 1,042 unique papers. After omitting those beyond the scope of the review, 61 full text articles were assessed for inclusion, with 44 articles deemed ineligible (not describing patient-reported experience *n* = 23, not focused on individuals living with CKD *n* = 11, conducted outside of COVID-19 timeframe *n* = 10). One article was a scoping review which included two eligible studies in the references. Seventeen were included in the final analysis.

### Characteristics of included studies

Table 2 contains the abbreviated data extraction for included studies (full version in supplementary materials).

**Table 2:** Papers meeting all search criteria (abbreviated version)

Author, date, Country	Setting	Number of patients Modality	Method of assessment (questionnaire, interview, other). tools validated (Y/N)	Theme	Findings	MMAT score
Alshaer et al. [23]	Kidney transplant recipients in UK	44 transplant recipients. 55% White, 55% male. Majority aged 50–64 years old	Feedback questionnaire	Remote consultation	88% of patients replied positively about their experience. Significant impact on the overall time saved, as well as convenient	[40%]
Androga et al. [16]	Nephrology Clinic in USA	3361 CKD outpatients (stages 1–5, treatment unspecified) Aged 47–75 years old, predominantly White	Patient Satisfaction Survey Not validated	Consultation over telephone/ video call	There was no difference in patient survey responses for the above when telehealth was compared to face-to-face visits	[80%]
Antoun et al. [20]	HHD and ICHD in UK	20 adult CKD patients (10 ICHD, 10 HHD) 70% male, Aged 52–77 years old	Semi-structured telephone interviews Not validated	QoL, Wellbeing and PA	QoL, Wellbeing, PA and Medical care all impacted ICHD negatively. HHD little change Focus on impact of shielding. Support offered was good	100%
Balson and Baharani [31]	UK dialysis unit	31 PD patients No further demographics	PREM with solely questions related to COVID-19. Not validated	Impact of COVID-19 on patient experience	Most patients felt kidney care had stayed the same 55% unable to shield as needing to attend hospital appointments 13% difficulties accessing medical assistance when needed	40%
Couzi et al. [28]	Kidney transplant recipients and candidates France	2112 Tx recipients, mean age 55 years old 487 candidates mean age 56 years old Both groups mostly male (around 60%)	Online survey. Not validated	Impact of COVID-19	Many (recipients: 19.6%; candidates: 54%) did not receive any information regarding COVID-19 from their transplant centre Issues raised about ability to work, accessing medication, transport. Shielding difficult due to need to attend hospital. 71% of candidates would prefer transplant surgery during COVID-19 surge rather than waiting	60%
Davis et al. [7]	Canadian Renal Units	141 Adults—HHD (98) and PD (43). 58% male, average age 53 years old	HD and PD Treatment Satisfaction Questionnaire, GAD7, PHQ-9, Illness Intrusiveness Ratings Scale, Family APGAR Questionnaire and The Self Perceived Burden Scale (All validated}	Treatment satisfaction and impact on mental health	No indication of a negative psychosocial impact from the pandemic on home-treated patients, despite the increased social isolation	40%
Huuskes et al. [22]	Kidney transplant recipients in Australia	53 transplant recipients. 53% female, 79% White, 47% aged 31–50 years old	Focus group on experience of telehealth	Remote consultation	Five themes identified, minimising burden, attuning to individual context, protecting personal connection and trust, empowerment and trust, and navigating technical challenges	[100%]
Ladin et al. [19]	Purposively sampled CKD patients, care partners, and clinicians. USA	30 patients older CKD patients (Stage 4,5 non-dialysis). 11 care partners 43% non-Hispanic Black ethnicity 67% female 73% over 75 years old	Semi-structured telephone interviews	Consultation over telephone/ video call	Tele-appointments though to be more convenient, less costly and more efficient—though worries about quality care, home diagnostics, loss of interpersonal connection and trust	[100%]
Lee et al. [18]	Renal clinic, Canada	235 CKD patients (treatment unspecified) 60% male, 92% White, 79% aged over 65 years old	Non-validated patient satisfaction survey	Consultation over telephone/ video call	Appreciation for safety and convenience of telephone consultation vs worries about lack of physical examination	[80%]
Malo et al. [30]	5 Dialysis centres in Canada	22 ICHD, 13 male and 9 female, mean age 60 years old, 14 White ethnicity, 4 Indigenous and 4 other ethnicity	Semi-structured interviews	Lived experience of CKD patients during COVID-19	Most patients did not report negative impacts on their care related to the pandemic. There were considerable disruptions of overall routine, including changes to transport and scheduling, particularly affecting indigenous patients	100%
Mazumder et al. [17]	Online. India	122 Tx Patients Mean age 43 years old 69% male	Rating scale of experience (0–10. 10 = best). Not Validated	Consultation over telephone/ video call	The mean patient satisfaction score for e-consults was 9.5	[20%]
McKeaveney et al. [24]	Two UK Renal Units	23 Adult Tx recipients, average age 49.4 years	Semi structured Interviews	Impact on mental health	Dealing with difficult conversations and managing ongoing fears of dialysis, distress and COVID-19	80%
O’Donnell, et al. [26]	UK Renal unit	200 Tx recipients 20–88 years old 59.6% male 84 White ethnicity, 24 South Asian, 6 African-Caribbean	Telephone interview. 4 scored questions -Overall lifetime happiness, overall lifetime satisfaction, momentary happiness, desire to change, 2 randomised groups—primed with question about COVID-19 and un-primed	Lifetime Satisfaction	Lifetime happiness, satisfaction, and momentary happiness higher in COVID-19 primed group. Non-primed higher desire to change	[80%]
Raina et al. [15]	62 centres across USA	400 paediatric patients (treatment unspecified, no other demographics)	Satisfaction survey. Not validated	Consultation over telephone/ video call	Overall, patients reported telemedicine visits as positive or neutral and with similar levels of satisfaction to in-person visits. Main issue was not being able to show consultants physical problems	[20%]
Scofano et al. [21]	Remote weekly monitoring of HHD in Brazil	17 HHD patients, mean age 80 years old, predominantly White (85%), male (64%) who completed Higher Education (76%)	Mixed methods including Telemedicine questionnaire—unvalidated	Consultation over telephone/ video call	64%—no difficulty using telemedicine, issues were due to data speed. Overall experience -76% rated positive Face-to-face visits considered a more complete care due to the possibility of undergoing a physical examination	[80%]
Sousa et al. [29]	Dialysis unit in Portugal	20 ICHD adults 55% male, average age 66.9 years old	Semi-structured telephone interviews	Patient experience—impact of COVID-19	Themes based on patient experience (a) psychosocial—impacts on family relationships, fear, increased distress. (b) negative impacts on disease and treatment-related health behaviours (c) positive impacts—personal growth and increased social support, and coping strategies	80%
Tse et al. [32]	Online survey. Recruited social media, kidney organisations and healthcare teams. UK	118 children & young persons (12–30 years) & parents. “Self-identified renal condition” HHD, ICHD, Tx, CKD 81% White	Mixed methods 7 open ended question survey Not validated	Experiences during COVID-19	Most common comments from CYA were that they felt they were missing out on work-related and educational opportunities (*n* = 14), missing family and friends (*n* = 9) and compared to their peers lived with more restrictions and “missing out on life (*n* = 8)”	80%

*CKD* chronic kidney disease, *HD* haemodialysis, *HHD* home haemodialysis, *ICHD* in-centre haemodialysis, *PD* peritoneal dialysis, *Tx* transplant, *GAD-7* Generalised anxiety disorder scale, *PHQ-9* patient Health Questionnaire-9, Family Adaptability, Partnership, Growth, Affection, and Resolve Questionnaire, *QoL* Quality of Life, *PA* Physical Activity, *CYA* Child and Young Adult

[] signifies that these articles relate to telemedicine only

### National setting

The articles cover a number of settings UK (*n* = 7), Canada (*n* = 3), USA (*n* = 2), Portugal (*n* = 1), Brazil (*n* = 1), France (*n* = 1), India (*n* = 1), Australia (*n* = 1).

### Study methodology

The study methods were varied and included questionnaires (*n* = 6), semi-structured interviews (*n* = 5), patient satisfaction surveys (*n* = 3), mixed method studies (*n* = 2), and focus group (*n* = 1).

### Treatment modalities

All treatment modalities were represented, with some studies including multiple treatment modalities—patients with a functioning transplant (*n* = 8), ICHD (*n* = 5), HHD (n = 3), PD (*n* = 2), CKD stages 3–5, not receiving kidney replacement therapy (*n* = 3), and CKD stage unspecified (*n* = 3).

### Patient diversity

Patient profile across the studies included mostly White (*n* = 9) males (*n* = 11) with seven studies not disclosing patient ethnicity, three of which did not include any patient demographics.

### Risk of bias

Nine of the 17 included articles related solely to patient experience of telemedicine. These were considered separately to those relating to patient experience of kidney care more globally. Overall, the quality of these studies as assessments of patient satisfaction with telemedicine was only fair, with MMAT scores ranging from 20 to 100% and with only five of the nine studies scoring 60% or more. Overall quality of the eight articles relating more directly patient experience of care were better, having MMAT scores ranging from 40 to 100%, with six scoring 60% or more.

### Themes

**Fig. 2:**
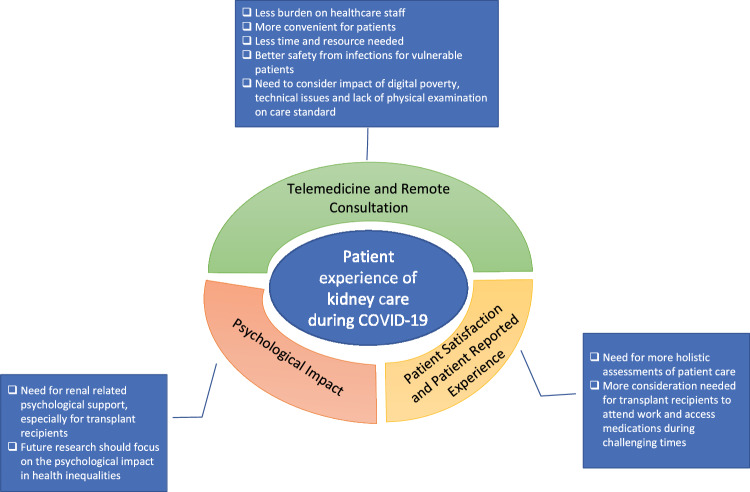
Summary of themes identified from the content analysis

After reviewing the findings from the included articles, and conducting a content analysis, the articles were summarised into three themes, Fig. 2 shows a summary of the themes, (1). Remote Consultation and telemedicine (*n* = 9), (2). Psychosocial Impact (*n* = 2) and, (3). Patient Satisfaction and Patient-Reported Experience (*n* = 6).

#### Theme 1: telemedicine and remote consultation

Use of telemedicine (telephone or video consultation) for healthcare consultations and communication increased during the pandemic. Telemedicine consultations were rated with similar levels of satisfaction as face-to-face consultations [15]. One study found significant differences in preferences according to the age of the patient, with older patients opting for face-to-face appointments. There were no differences in relation to ethnicity and gender [16]. A recurring issue with telemedicine was the inability to have physical issues examined by the consulting clinician [15–17].

Lee et al. [18] evaluated patient satisfaction in 235 people with CKD who had attended face-to-face outpatient consultations before the pandemic and had telemedicine appointments during it. Patients reported feeling less anxious about telemedicine appointments since they avoided having to attend hospital, and thus the need to travel, find parking, and endure clinic waits. They were more willing to meet in-person when symptoms worsened or changes in clinical care were warranted. Most nephrologists felt telephone consultations increased accessibility, especially for elderly patients and those with physical disabilities. Disadvantages included lack of clarity of information, reduced opportunities to develop connections and trust with nephrologists, lack of opportunity for physical examination, and the impersonal nature of telephone consultation. Younger patients and those with minority ethnic backgrounds were under-represented in the sample [18].

A similar study [19] involved 30 older patients (70 years and above) with CKD stages 4 to 5, and 11 care partners. Some patients worried about quality of care and home diagnostics, *“I don’t think it’s a good idea to try to diagnose people over the telephone…Your machine may not be as good as the ones at the doctor’s office, and you may be getting a wrong result.”* Others felt a loss of interpersonal connection and trust. However, telemedicine appointments were also thought to be more convenient, less costly, and more efficient. Patients of Black ethnic origin expressed more concern, *“[With telehealth] I feel like I'm on my own…I’m looking for help. And I wasn’t getting it that much.”* Overall, while telehealth reduced barriers to care for some older adults, more support is needed for those with limited English proficiency, hearing loss, and limited access to internet and technology [19].

The impact of telemedicine on physical activity, wellbeing, and quality of life (QoL) has also been studied [20]. Ten patients receiving haemodialysis were interviewed after the first wave of COVID-19. Those receiving HHD had felt little impact on their wellbeing, physical activity and QoL, whilst those receiving ICHD felt a significant negative impact across these domains [20].

Scofano et al. [21] studied the use of telemonitoring to maintain and improve relationships between clinical staff and patients during the pandemic. Patients on assisted HHD (mean age 80) who had received remote weekly monitoring were asked “What would you tell a friend about your experience with telemonitoring?”. Sixty-four percent experienced no difficulty using telemedicine. The main issue identified was related to data transfer speed. Most fully or partially agreed that telemonitoring helped monitor their treatment, increased communication with the clinician and improved their understanding of clinical instructions. Overall, 76% rated their experience positively. However, face-to-face visits with the doctor were considered a more complete form of care incorporating the possibility of physical examination [21].

Huuskes et al. conducted 5 focus groups with kidney transplant recipients to identify their perceptions of the use of telehealth during COVID-19. They concluded 5 themes which impacted the experience of telehealth, these included minimising burden, attuning to individual context, protecting personal connection and trust, empowerment and trust, and navigating technical challenges [22]. Alshaer et al. recognised the importance of continuing to monitor kidney transplant recipients during the pandemic but in a way that would keep them safe. The implementation of telephone and video consultations was rapid; Alshaer et al. collected feedback from 44 patients who were receiving virtual consultations to share their experience [23]. At the end of each video appointment, the patient would receive an optional feedback questionnaire about their experience of their virtual appointment. Sixty-six percent of those who responded said their experience was no different from a face-to-face consultation, with only 9% scoring their virtual experience as poorer [23]. Highlighted benefits of virtual consultation provided in the feedback were convenience, saving time overall and not requiring time off work to attend; safer—77% recognised virtual consultations were safer during the pandemic; greater comfort as they could have the review at home; more cost efficient as they were not required to pay for transport or parking. The study concluded that virtual consultations for individuals with a functioning transplant could replace face-to-face consultations in the majority of cases, where patients do not necessarily need to be examined [23].

#### Theme 2: psychosocial impact

McKeaveny et al. [24] examined the lived experiences of kidney transplant recipients. Twenty-three adults took part in semi-structured interviews between June and October 2020. Two key themes emerged, “dealing with difficult conversations” and “managing ongoing fears of dialysis, distress, and COVID-19”. Communication with family and friends, and with non-renal healthcare professionals, including those supporting mental health, was often undermined by the perception that people could never fully grasp the patient’s situation. There was also a perception of immense pressure from healthcare professionals to explore living donation with family and friends. The transplant journey was described as often dominated by feelings of instability. Dialysis, though life-sustaining, was often perceived as associated with rapid decline in functionality and wellbeing, to be avoided at all costs. Whilst understanding the importance of shielding during the pandemic, many described not seeing children and partners, which living away from the family home entailed, as leading to poorer mental health including increased anxiety, depression, and paranoia. These findings highlight the need for psychological support for this group [24]. In contrast, those receiving home therapies projected feelings of safety, use of flexible schedules to maintain autonomy and independence and the capacity to continue working during a period of economic instability [25]. There were clear differences in the psychosocial impact of COVID-19 on people on different modalities. The impact of age, gender, and ethnicity on psychological state has not been evaluated.

Some studies [29, 30, 32] that covered under the theme ‘patient satisfaction’ and patient-reported experience’ during the pandemic, also covered aspects of psychological impact. Sousa et al. identified 4 themes which related to psychosocial elements of their experience including fear and coping strategies [29]. Malo et al. covered multiple aspects of patient experience, with patients revealing they feared infecting their family members [30]. Tse et al. found similar results in children and young adults [32].

#### Theme 3: patient satisfaction and reported experience during COVID-19

During shielding, individuals living with a kidney transplant were asked, during a telephone interview, to score four questions about lifetime and momentary happiness, satisfaction, and the desire to change [26]. Half were randomised to be primed by being asked how they were feeling about COVID-19 prior to the interview questions. The others were un-primed. The primed group scored significantly higher on questions about satisfaction and happiness and fewer desired change [26]. This says little about their satisfaction during COVID-19 but does indicate how priming, by referring to negative circumstances (in this case the pandemic), can enhance responses about momentary happiness and global satisfaction, presumably by providing information which can be used to attribute and partially offset the effect of momentary mood on global life satisfaction [27]. This has important implications for measuring patient satisfaction more broadly.

A French study, performed during the first wave of COVID-19 [28], examined preventive behaviours and concerns, information sources, and rates of infection in transplant recipients and those on transplant waiting lists. A high proportion of recipients (69.4%) and those waiting (80.1%) left their home during shielding, but mainly for healthcare purposes. A significant number reported not receiving any information regarding COVID-19 from their transplant centre (recipients: 20% vs. those waiting: 54%; *P* < 0.001). Their main sources of information were television and the internet. Transplant recipients more frequently thought that the pandemic affected their ability to work (33 vs. 23%; *P* < 0.001), to access medications (27 vs. 18%; *P* = 0.002) and access the hospital (24 vs. 18%; *P* = 0.002) than those wait-listed. Seventy-one percent of wait-listed patients would prefer to undergo transplantation as soon as possible rather than wait for the pandemic to abate.

A Portuguese study, carried out in the first wave of the pandemic, used semi-structured interviews to examine the experience of patients receiving ICHD [29]. Four themes emerged, there were negative psychosocial impacts on patients and on family relationships. Patients reported increased fear, distress and worry about contracting COVID-19. There were impacts on treatment-related health behaviours, including difficulties managing dietary restrictions and reduced physical activity due to shielding. Some, however, experienced personal growth and reported increased social support. Others developed coping strategies and actively sought opportunities to enhance support [29]. A Canadian study of individuals receiving ICHD, carried out later in the pandemic, deploying semi-structured interviews [30] found that most patients did not report negative impacts on their care. They did report considerable disruptions of overall routine, including changes to transport and scheduling, particularly affecting indigenous patients. There were also concerns about contracting the virus, particularly from healthcare workers, of infecting the family, and having to shield and stay away from family. Future research focused on the impact of health emergencies on marginalised populations is warranted [30].

A UK study, focused on patients receiving PD [31], used a bespoke questionnaire to examine patients’ experience of shielding, of accessing dialysis and general medical care, and thoughts and feelings about the future. Most individuals felt that their care had not changed. Most (90%) were aware of shielding and had received a letter to advise them of this, but 55% reported being unable to shield completely because they needed to attend hospital appointments and 13% recorded difficulties accessing medical assistance when needed. Sixty percent felt negatively or ambivalently about the future. These findings imply the need for better support for this group.

A UK survey deployed several open questions to examine experiences during COVID-19 of children, young adults (median age 21) with chronic kidney conditions and their parents [32]. The most common children, young adults’ comments related to inability to engage with educational and work-related opportunities. They reported missing family and friends and ‘missing out on life’—perceiving greater restrictions than their peers [32]. Shielded patients reported being more vigilant about COVID-19 rules, and to feeling more protected yet more isolated than non-shielding patients. Many felt they had received limited information or mixed messages. This was echoed by parents. Only a minority reported that they would like more support from an educational institution (children, young adults 14%, parents 20%) or support to reduce worries (children, young adults 21%, parents 31%).

## Discussion

This review provides a first attempt to synthesise published reports on the impact of COVID-19 on patient experience of kidney care. Most focused on patient satisfaction with remote consultations. Patients were generally satisfied, appreciating the convenience and safety but were worried about compromised clinical rigour and skipped clinical examinations. Digital literacy and technical difficulties posed problems particularly for older patients and patients identifying as minority ethnic backgrounds. A scoping review [33] of eight articles, explored patient perspectives of remote consultations both during COVID-19 and before. Patients with kidney disease, overall, were satisfied with remote services, listing convenience and increased involvement in their care as benefits. Barriers included technical difficulties, digital literacy, and loss of interpersonal communication. Concerns mainly related to privacy and confidentiality, lack of physical examination and non-verbal cues being missed. Pre-requisites for success included existing patient-practitioner relationships and access to digital technologies [33]. This review addresses only one area of patient experience of care.

Psychosocial impact was a theme also capturing an aspect of patient experience of care. Individuals with functioning transplants felt insecure and dreaded returning to dialysis. Shielding was a source of anxiety and depression. The need for psychological support was evident. Those on home therapies felt safer. There was little data on people receiving ICHD nor on the impact of health inequalities, such as the psychological impact on older versus younger patients. Differences in patient experience of care between modalities are evident from this review, particularly in relation to the psychological impact of COVID-19, but these differences are often inferred from comparison of single modality studies of aspects of patient experience. Direct comparisons between modalities of holistically assessed experience of care are lacking. A number of studies discussed under the theme ‘patient satisfaction and patient-reported experience’ also covered aspects of psychological impact, such as support offered, and mental health considerations [29, 30, 32].

The remaining studies attempted a more holistic approach to assess patient experience, though most addressed patient satisfaction with care relating to different modality groups making direct comparison difficult. Transplant recipients felt an impact on their ability to work and to access medications. Those receiving ICHD had mixed views perhaps relating to the phase of the pandemic of the studies; those in later studies experienced fewer negative effects. Most patients receiving PD felt little change, though many were pessimistic about the future [31]. Younger patients commonly felt they were missing out on life [32].

The study has a number of limitations. Rapid reviews are useful for examining current evidence related to a specific question, however by their nature they only focus on peer reviewed articles, written in the authors’ first language; in this case only articles written in English were included for screening. Although a strength of rapid review is that it can evaluate current research in a short timeframe, this can also be argued as a weakness of the review due to the limited number of articles included for screening.

The risk of bias assessment indicates that the overall quality of the articles was only fair, particularly those relating to telemedicine. Albeit these do not directly assess the aims of this review, which call for a more holistic assessment of patient experience of care, the articles were included as telemedicine played an important role in patients’ kidney care during the pandemic. The quality of the articles assessing patient experience of care more directly was a little better, though with many focussing on a single modality.

Furthermore, patient satisfaction with an aspect of management is very different from overall patient experience of care. This is best conceived as the individual’s perception of the range of interactions they have with their healthcare system [34]. As such, it is an indicator of healthcare quality. Patient satisfaction measures reflect the extent to which the individual is happy with care; the degree to which care meets their expectations. It is less reliable as a measure of healthcare quality [34]. This is a significant limitation of this study in that most of the included studies lack a holistic view of the patients’ experience of care.

## Conclusion

Patient experience of kidney care was significantly impacted by the pandemic. Current literature focusses on satisfaction with telemedicine as a replacement for hospital consultations, and patient satisfaction with other aspects of care. There is little emphasis on holistic assessment of patient experience, less on how this differed between modalities and even less on differences in care experiences driven by the wider determinants of health inequalities. Such assessments could be helpful in optimising care provision across the management spectrum of CKD and in planning for future disruptions in care, such as war and natural disasters.

## Registration of protocol

PROSPERO 2022 CRD42022363306

Available from: https://www.crd.york.ac.uk/prospero/display_record.php?ID=CRD42022363306.

### Supplementary Information

Below is the link to the electronic supplementary material.

Supplementary file1 (DOCX 30 KB)

## Data Availability

The datasets during and/or analysed during the current study available from the corresponding author on reasonable request.
