# Supplementary material for: Impact of COVID-19 on patient experience of kidney care: a rapid review
Source: J Nephrol. 2023 Dec 20;37(2):365–78. doi: 10.1007/s40620-023-01823-5 (PMC11043167; doi:10.1007/s40620-023-01823-5)
Supplement: Supplementary file 1 — Supplementary file1 (DOCX 30 KB) [file 40620_2023_1823_MOESM1_ESM.docx]

# Supplementary Materials

Papers meeting all search criteria (full version)

| **Author, date,**  **Country**  **Title** | **Purpose of Study** | **Setting** | **Number of patients.** | **Method of Assessment (Questionnaire, Interview, other)** | **Assessment (WR to COVID-19)** | | **Theme** | **Main Findings** | | **Limitations** | **MMAT Score** |
| --- | --- | --- | --- | --- | --- | --- | --- | --- | --- | --- | --- |
|  |  |  | **Treatment Modality** | **Tools**  **Validated (Y/N)** | **Pre-** | **Post-** |  | **Pre-** | **Post-** |  |  |
| Alshaer, Teles, Imedi,  UK  2020  Virtual renal transplant clinics: patients' and clinicians' experiences | To determine patient experience of remote consultation | Kidney transplant recipients in UK | 44 transplant recipients. 55% White, 55% Male. Majority aged 50-64 years old. | Feedback questionnaire |  | Following video consultation patients were given an option to complete a feedback questionnaire regarding their experience | Remote consultation |  | 88% of patients replied positively about their experience. Significant impact on the overall time saved, as well as convenient. | Focussed only on one treatment type. | [40%] |
| McKeaveney, Noble, Courtney, Griffin, Gill, Johnston, Maxwell, Teasdale, Reid  UK  2022  Dialysis, Distress, and Difficult Conversations: Living with a Kidney Transplant | The aim of this study was to develop an in-depth understanding of the lived experiences of kidney transplant recipients. | Two regional UK Nephrology units | 23 Adult transplant recipients, average age 49.4 years. | Semi structured Interviews |  |  | Psychosocial | 1. Dealing with difficult conversations   Kidney transplantation is associated with extreme physiological and psychological  stressors | 1. Managing ongoing fears of dialysis, distress and COVID-19   The COVID-19 pandemic further demonstrates the urgent need to provide psychological support to this patient population | results are not transferable  to the entire population of participants living with a kidney transplant | 80% |
| Davis, Alqarni, McGrath-Chong, Bargman, Chan  2021  Canada  Anxiety and psychosocial impact during coronavirus disease 2019 in home dialysis patients | The aim of this study is to describe levels of anxiety and quality of life during the COVID-19 pandemic among home dialysis patients | Canadian Home dialysis patients | 141 Adults HHD (98) and peritoneal dialysis (43). 58% Male, average age 53 years old. | Haemodialysis and Peritoneal Dialysis Treatment Satisfaction Questionnaire, Generalized Anxiety Disorder 7 Item (GAD7) Scale, Patient Health Questionnaire (PHQ-9), Illness Intrusiveness Ratings Scale, Family APGAR Questionnaire and The Self Perceived Burden Scale  {All measures are validated} |  | Questionnaires during pandemic | Psychosocial |  | Satisfaction quite high overall for majority of patients.  “a feeling of safety from COVID-19 with no travel to in-centre appointments”, “a flexible schedule that maintained autonomy and independence while also allowing patients to continue working during a period of potential economic instability”, “the support of family, children and a familiar environment”, “continuing to have a liberated diet and the opportunity to exercise” and “the opportunity to maintain social activities and lifestyle practices.” | A selection bias exists in home dialysis patients who sometimes have a higher capacity to cope | 40% |
| O’Donnell, Wilson, Bosch & Borrows  2020  UK  Life satisfaction and happiness in patients shielding from the COVID-19 global pandemic: A randomised controlled study of the ‘mood as information’ theory | to inform health care providers of the impact of bringing the pandemic to salience during life satisfaction evaluations, assessing whether this ‘prime’ results in increased or decreased reports of satisfaction which are derived unconsciously. | Renal Transplant Department in a tertiary centre in the United Kingdom.  Queen Elizabeth Hospital, Birmingham | 200 Tx recipients  20-88 years old  59.6% male  84 White ethnic, 24 South Asian, 6 African-Caribbean | Telephone interview  4 questions scalar (10)  -Overall lifetime happiness  - desire to change  - overall lifetime satisfaction  - momentary happiness  2 groups  COVID-19 primed (asked about COVID-19) before 4 questions and non-primed |  | During May 2020 “height of shielding” | Satisfaction |  | Significant differences between 2 groups for all 4 questions  Lifetime happiness, satisfaction and momentary happiness higher in COVID-19 primed group.  Non-primed higher desire to change | Just transplant  Focus on mood as information theory rather than kidney care | [80%] |
| Lee, Ramondino, Gallo, Moist  2021  Canada  A Quantitative and Qualitative Study on Patient and Physician Perceptions of Nephrology Telephone Consultation During COVID-19 | To evaluate patient and physician perspectives on the key advantages and disadvantages of telephone consultations in a nephrology out-patient clinic setting | General nephrology clinic and a multidisciplinary kidney care clinic in London, Ontario, Canada | 235 CKD patients (treatment unspecified)  60% male, 92% White, 79% aged over 65 years old | Non-validated patient satisfaction survey | Had attended face-to-face consultation | Had telephone consultation  Patient satisfaction survey, Likert scale with 2 additional free text questions | Patient Satisfaction and Telemedicine |  | Themes regarding advantages of telephone consultation included the perceptions of safety and convenience of telephone consultation as participants felt less anxious about having to enter the hospital during the pandemic and favoured telephone consultation due to reduced need for travel, parking, and waiting at the clinic. However, participants were more willing to meet in-person when their symptoms become more severe or a change in medical needs was warranted. Themes regarding disadvantages had to do with lack of clarity of information, developing connections and trust with nephrologists, lack of the physical examination, and the impersonal component of telephone consultation. | One location, not generalisable | [80%] |
| Antoun, Brown, Jones et al  2021  UK  Understanding the Impact of Initial COVID-19 Restrictions on Physical Activity, Wellbeing and Quality of Life in Shielding Adults with End-Stage Renal Disease in the United Kingdom Dialysing at Home versus In-Centre and Their Experiences with Telemedicine | The aim of this study was to investigate how these restrictions and the transition to an increased reliance on telemedicine within clinical care of people living with kidney disease impacted the physical activity (PA), wellbeing and quality of life (QoL) | adults dialysing at home (HHD) or receiving in-centre haemodialysis (ICHD) in the UK | 20 adult CKD patients (10 ICHD, 10 HHD)  70% Male, Aged 52-77 years old | Semi-structured telephone interviews  Not validated |  | Interviews post COVID  Questions on how changed to kidney care affected QoL, physical activity, Wellbeing and medical care | QoL, Wellbeing and PA  Telemedicine |  | QoL, Wellbeing, PA and Medical care all impacted  ICHD negatively impacted  HHD little change  Focus on impact of shielding. Support offered was good | Only consider HHD and ICHD | 100% |
| Sousa, Ribeiro, Costa et al  2020  Portugal  Being on hemodialysis during the COVID-19 outbreak: A mixed-methods’ study exploring the impacts on dialysis adequacy, analytical data, and patients’ experiences | This study aimed to explore the impacts of the COVID-19 pandemic in non-COVID-19 patients with ESRD undergoing in-center hemodialysis (HD). | One dialysis unit in Portugal | 20 ICHD adults  55% male, average age 66.9 years old | Mixed methods  Quant data to show adequacy of dialysis  Qual data from semi-structured telephone interviews | Feb 2020  Clinical outcomes of dialysis from patient medical records | April 2020  (during lockdown)  Clinical outcomes of dialysis from patient medical records + qual interview | Dialysis accuracy and patient experience – impact from COVID-19 | Patient records of dialysis adequacy markers  (interdialytic weight gain, urea, urea reduction ratio, Kt/V, and minutes per dialysis session)  Analytical markers  (potassium, sodium, phosphorus, calcium, haemoglobin, haematocrit, erythrocytes, mean corpuscular volume, mean corpuscular haemoglobin, mean corpuscular haemoglobin concentration, red cell distribution width, platelets, white blood cells, total proteins, albumin, and normalized protein catabolic rate) | Overall results suggested that dialysis adequacy and serum albumin levels decreased significantly at T2, while phosphorus levels increased  Themes based on patient experience  (a) psychosocial negative impacts (impacts on family relationships, fear, increased distress).  (b) impacts on disease and treatment-related health behaviours (difficulties managing dietary restrictions during the lockdown and diminished physical activity)  (c) positive impacts (personal growth and increased social support), and (d) coping strategies (engaging in exercise and seeking support). |  | 80% |
| Balson and Baharani  2021  UK  Peritoneal dialysis patients – the forgotten group in the coronavirus pandemic | Our aim was to better understand how patients on PD have been affected by the pandemic thus far, and how renal services could better support them going forward. | Tertiary Birmingham dialysis unit | 31 PD Patients  No further demographics | PREM with solely questions related to COVID-19  Not Validated |  | PREM | Impact of COVID-19 on PD Patients |  | Most patients felt kidney care had stayed the same  90% aware of shielding and received letter to shield but 55% unable to shield as needing to attend hospital appointments  13% recorded difficulties accessing medical assistance when needed |  | 40% |
| Raina, Nair, Yap et al  2021  USA  Survey of Telemedicine by Pediatric Nephrologists During the COVID-19 Pandemic | Understand experience of telemedicine from patient and provider | 62 centres across North America | 400 patients (treatment unspecified, no other demographics) | Satisfaction survey  Not validated |  | Survey data collect March to August 2020 | Telemedicine |  | Overall, patients reported telemedicine visits as positive or neutral and with similar levels of satisfaction, compared with in-person visits.  Only issue with telemedicine was not being able to show consultants physical problems |  | [20%] |
| Malo, Affdal, Blum et al  2022  Canada  Lived Experiences of Patients Receiving Hemodialysis during the COVID-19 Pandemic: A Qualitative Study from the Quebec Renal Network | The objective of this study was to gain a better understanding of the scope and magnitude of the effects of the pandemic on the lived experience of patients receiving in-center hemodialysis. | 5 haemodialysis centres in Montreal, Canada | 22 ICHD, 13 male and 9 female, mean age 60 years old, 14 White ethnic, 4 Indigenous and 4 other ethnicity | Semi-structured interview  Not validated |  | Interviews conducted between November 2020 and May 2021 | Lived experience of CKD patients during COVID-19 |  | COVID-19 was not associated with negative effects on patients' medical care.  Themes include  -change in routine  -change in transportation routine  -changing to HHD  -concerns regarding infection  -risk management  -fear of infecting family  -isolation  -interaction with healthcare workers |  | 100% |
| Scofano, Monteiro, Motta  2022  Brazil  Evaluation of the experience with the use of telemedicine in a home dialysis program—a qualitative and quantitative study | The main objective of this study was to evaluate whether telemedicine provided through telemonitoring can improve the ongoing relationship between the doctor, the nurse and the patient. | Remote weekly monitoring of HHD patients in Rio de Janeiro | 17 HHD patients, mean age 80 years old, predominantly White (85%), Male (64%) who completed High Education (76%) | Mixed methods Telemedicine questionnaire -not validated |  | Telemedicine monitoring via Zoom weekly from June 2020 to Jan 2021  “What would you tell a friend about your experience with telemonitoring?” | Telemedicine |  | 64% no difficulty with ease of using telemedicine, issues were due to data speed.  Most of the patients fully or partially agreed that telemonitoring helps in monitoring their treatment, increases communication with the doctor and increases their understanding of medical instructions.  Overall experience, 76% rated positive  Face-to-face visits with the doctor were considered a more complete form of care due to the possibility of undergoing a physical examination |  | [80%] |
| Androga, Amundson, Hickson et al  2022  England  Telehealth versus face-to-face visits: A comprehensive outpatient perspective-based cohort study of patients with kidney disease | We aimed to use objective data to study patients’ perspectives on outpatient nephrology care received via telenephrology (phone and video) versus face-to-face visits. | Nephrology and Hypertension Clinic in Rochester | 3361 CKD Outpatients (stages 1-5, treatment unspecified)  Aged 47-75 years old, predominantly white | Patient Satisfaction Survey  Not validated |  | Surveys distributed March to July 2020  Using a five-point Likert scale ranging from 1 = very bad to 5 = very good, surveyed patients expressed their perspectives with regard to access to their nephrologist, how they related to their care provider, and when relevant–their opinions on the telemedicine technology–and their overall assessment of the care they received during the nephrology visit. | Telemedicine |  | There was no significant difference in patient survey responses for the above when telehealth was compared to face-to-face visits. A large majority of patients selected a Likert score of at least 4 or 5 for all survey questions |  | [80%] |
| Couzi, Manook, Caillard et al  2021  France  Impact of COVID-19 on kidney transplant and waiting list patients: Lessons from the first wave of the pandemic | to elicit and understand the behaviours, concerns, and priorities of kidney transplant recipients and waiting list candidates at the end of the first wave of the pandemic in France | French kidney candidates and recipients were contacted directly by the French patient association France Rein or by transplant centers | 2112 Tx recipients, mean age 55 years old  487 candidates mean age 56 years old  Both groups mostly male (around 60%) | Online survey  Not validated |  | May 2020  Patients completed the survey | Transplant patients impact from COVID-19 |  | 69.4% of transplanted and 80.1% of candidates left their home during shielding, mostly due to healthcare purposes  A significant number of patients did not receive any information regarding COVID-19 from their transplant center (recipients: 19.6% vs. candidates: 54.0%; P < 0.001), with the main source being television then internet.  Recipients more frequently thought that the COVID-19 pandemic could affect their ability to work (33% vs. 22.8%; P < 0.001), their ability to get medications (26.6% vs. 17.5%; P = 0.002), and their transportation to the hospital (24.4% vs. 17.7%; P = 0.002) than candidates  Among candidates, 71% preferred to undergo transplant as soon as possible, 19.5% preferred to wait until COVID-19 had left their community, and 9.4% were not sure what to do |  | 60% |
| Ladin, Porteny & Perugini  2021  USA  Perceptions of Telehealth vs In-Person Visits Among Older Adults With Advanced Kidney Disease, Care Partners, and Clinicians | o identify patient, care partner, and nephrologists’ perceptions of the patient-centeredness, benefits, drawbacks of telehealth compared to in-person visits | purposively sampled patients, care partners, and clinicians in Boston, Massachusetts; Chicago, Illinois; Portland, Maine; and San Diego, California. | 30 patients older CKD patients (Stage 4,5 non-dialysis). 11 care partners  43% non-Hispanic black ethnic  67% Women  73% over 75 years old | Semi-structured telephone interviews |  | Interviews conducted between august and December 2020 | Telehealth |  | Patients worried about quality care and home diagnostics. Patient 33 shared: “I don't think it’s a good idea to try to diagnose people over the telephone…Your machine may not be as good as the ones at the doctor’s office, and you may be getting a wrong result.”  Also, loss of interpersonal connection and mistrust  However, patients also reported for tele-appointments to be more convenient, less costly and more efficient  These findings suggest that while telehealth reduces barriers to care for some older adults, greater resources are needed to support many older adults with chronic illness, including limited English proficiency, hearing loss, and those with limited access to internet and technology. | Limitations include recall bias and underrepresentation of Hispanic patients and non-English speakers, | [100%] |
| Tse, Darlington, Tyerman et al  2021  UK  COVID-19: experiences of lockdown and support needs in children and young adults with kidney conditions | To investigate the experiences, information needs, decision-making and support needs of children and young adult (CYA) patients or their parents during COVID-19 | Online across the UK recruited through social media, kidney organisations and healthcare teams | 118 children & young persons (12-30 years) & parents. “Self-identified renal condition”  HHD, ICHD, Tx, CKD  81% White | Mixed methods  7 open ended question survey  Not validated |  | Online survey distributed between May and June 2020 | CYA experiences during COVID-19 |  | 40% of CYA worried about the virus  most common comments from CYA was they felt they were missing out on work-related and educational opportunities (n = 14), missing family and friends (n = 9) and compared to their peers they lived with more restrictions and were missing out on life (n = 8). | We did not measure access to virtual clinics or urban/rural geography which may have influenced respondents’ experience of healthcare. The use of social media to distribute and access the study may have also limited access to English-speaking individuals with compatible devices/smartphones | 80% |
| Mazumder, Gulati, Shehwar, Sengar  2022  India  A single-center prospective observational study evaluating telemedicine for kidney transplant patients in the Coronavirus disease-19 pandemic: Breaking the access barrier | to assess the feasibility, acceptability, and effectiveness of telemedicine services for regular follow-up of kidney transplant patients as well as for triaging patients for admission | Online across India | 122 Tx Patients  Mean age 43 years old  69% Male | patients were asked to rate their experience on a scale of 0 to 10 (0 being not satisfied and 10 being fully satisfied).  Not Validated |  | March to June 2020  Question asked electronically after telemedicine consult | Telehealth |  | The mean patient satisfaction score for e-consults was 9.5. | Study needs to be validated in a larger cohort  Little data on satisfaction and patient experience | [20%] |
| Huuskes, Scholes-Robertson, Guha, Baumgart, Wong, Kanellis, Chadban, Barraclough, Viecelli, Hawley, Kerr, Coates, Amir, Tong  2021  Australia  Kidney transplant recipient perspectives on telehealth during the COVID-19 pandemic | The aim of this study was to describe the perspectives of kidney transplant recipients on telehealth during the COVID-19 pandemic. | Transplant recipients in the Australia | 53 transplant recipients, 53% women, 47% received their transplant from deceased recipients, 79% White ethnic heritage, 47% aged 31-50 years old | Focus group |  | Each participant attended one of five one-hour focus groups, convened in August 2020 using ZOOM videoconferencing. Each group consisted of between 5 and 10 participants. | Remote consultation |  | minimising burden, attuning to individual context, protecting personal connection and trust, empowerment and trust, and navigating technical challenges | Focus on transplant recipients only. | [100%] |
|  |  |  |  |  |  |  |  |  |  |  |  |

[] signifies that these articles relate to telemedicine only
